# Type II heparin-induced thrombocytopenia in a maintenance hemodialysis patient: A case report and literature review

**DOI:** 10.1097/MD.0000000000044821

**Published:** 2025-09-26

**Authors:** Xiaming Zhang, Mi Zhou, Ping Wu, Yingji Chen, Juzhen Yan

**Affiliations:** a Department of Nephrology, The Affiliated Hospital of Hangzhou Normal University, Hangzhou, Zhejiang Province, People’s Republic of China.

**Keywords:** argatroban, heparin-induced thrombocytopenia, low molecular weight heparin, maintenance hemodialysis, rivaroxaban

## Abstract

**Rationale:**

Heparin-induced thrombocytopenia (HIT) is a rare but severe immunologically mediated drug reaction characterized by a significant decrease in platelet count following exposure to heparin, accompanied by hypercoagulability or thrombosis. Severe cases can present as acute allergic reactions, which can be life-threatening. We report a rare case of type II HIT in a hemodialysis patient, initially presenting with arteriovenous fistula thrombosis, and review the literature to share strategies for early recognition and management.

**Patient concerns:**

We present a case involving a 69-year-old female patient who experienced significant thrombocytopenia, thrombosis of an arteriovenous fistula, and an acute hypersensitive reaction after being exposed to low molecular weight heparin during the initiation of dialysis.

**Diagnoses:**

She scored 8 points on the 4T scoring system, and her HIT immunoglobulin G–specific antibody test returned positive, confirming a diagnosis of HIT.

**Interventions:**

All heparin-based anticoagulants were discontinued, and argatroban was introduced as a substitute.

**Outcomes:**

The patient’s platelet count recovered to near baseline levels within approximately 1 week after stopping heparin, and no further symptoms related to acute systemic reactions occurred. The patient was eventually transitioned to novel oral anticoagulants and then discontinued argatroban. Her dialysis has been proceeding smoothly so far, and there have been no further occurrences of arteriovenous fistula thrombosis.

**Lessons:**

Our experience emphasizes the importance of closely monitoring clinical manifestations and platelet count changes during the first 1 to 2 weeks following the initiation of dialysis. If there is a significant drop in platelet count, it is crucial to consider the possibility of HIT in conjunction with the patient’s medical history and clinical presentation. In addition, hemodialysis can be safely performed using only oral rivaroxaban.

**Key points**

- Close monitoring of platelet count changes is required 5 to 10 days after the first hemodialysis session.
- In patients undergoing hemodialysis with standard heparin or low molecular weight heparin anticoagulation, if there is a significant decrease in platelet count within 1 to 2 weeks, along with allergic reactions, new thrombus formation, or increased clotting in dialysis circuits, it is necessary to investigate the possibility of HIT.
- The 4T score combined with HIT antibody testing and/or platelet function tests can help diagnose HIT.
- Once HIT is confirmed, heparin-containing drugs must be discontinued immediately. For anticoagulation during hemodialysis, alternatives to heparin include argatroban injection, nafamostat mesylate injection, and sodium citrate injection.
- Patients with concomitant thrombotic events can be treated with oral warfarin sodium tablets or novel oral anticoagulants, such as rivaroxaban 15 mg twice daily, with dose reduction to 20 mg once daily after platelet count returns to normal or after 3 weeks.
- Rivaroxaban taken alone for anticoagulation can also allow hemodialysis to proceed smoothly.

## 1. Introduction

Heparin-induced thrombocytopenia (HIT) is an infrequent yet severe immune-mediated disorder characterized by a substantial reduction in platelet count exceeding 50% from baseline values. This reduction typically occurs 5 to 10 days after starting heparin therapy, and it is uncommon for the absolute platelet count to drop below 20 × 10^9^/L.^[1]^ It is associated with a prothrombotic state and the presence of heparin-dependent, platelet-activating IgG antibodies.^[2]^ The incidence of HIT is estimated to be between 0.1% and 5.0% among patients receiving heparin therapy. Rates for unfractionated heparin range from 1.0% to 5.0%, while those for low molecular weight heparin range from 0.1% to 1.0%.^[3]^ The highest incidence is observed in female patients, especially after orthopedic or cardiac surgeries that commonly use low molecular weight heparin anticoagulation.^[4,5]^ Unfractionated heparins and low molecular weight heparins are widely used for anticoagulation during hemodialysis treatment, as they prevent blood from clotting in the extracorporeal circuit.^[6]^ Herein, we report a case of HIT with significant thrombocytopenia, arteriovenous fistula (AVF) thrombosis, and an acute hypersensitive reaction after receiving maintenance hemodialysis treatment.

## 2. Case report

**Figure 1.**
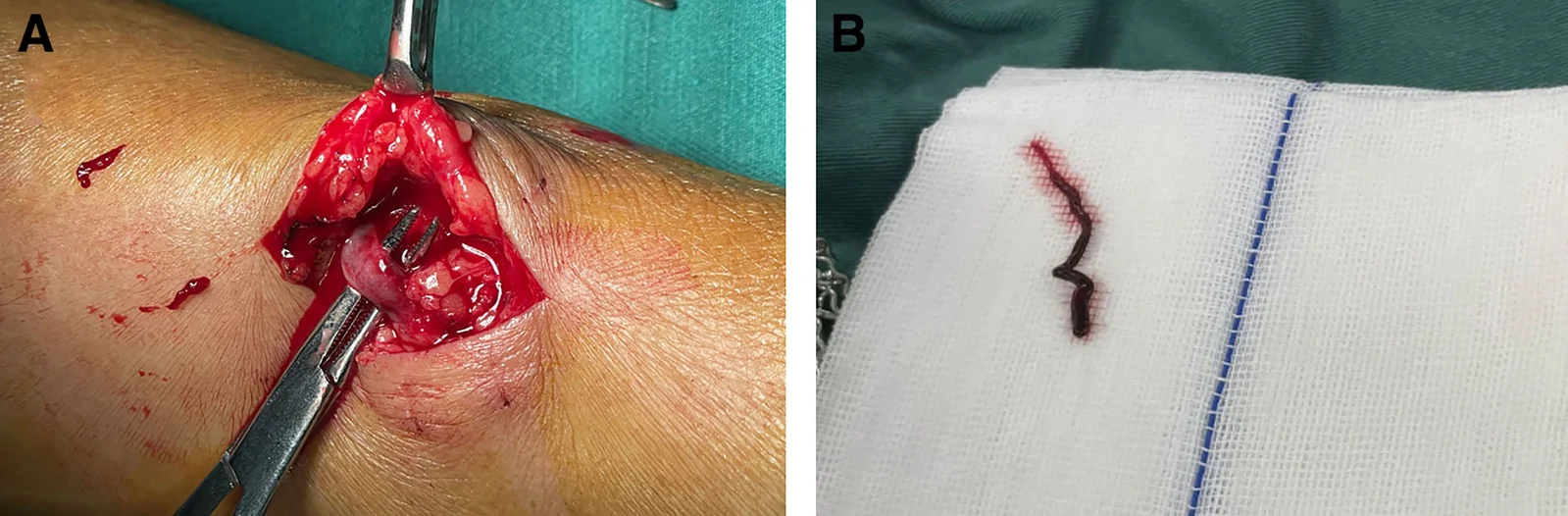
(A) The cephalic vein of the arteriovenous fistula in the left forearm with thrombosis and occlusion. (B) The thrombus that is removed from the cephalic vein.

**Figure 2.**
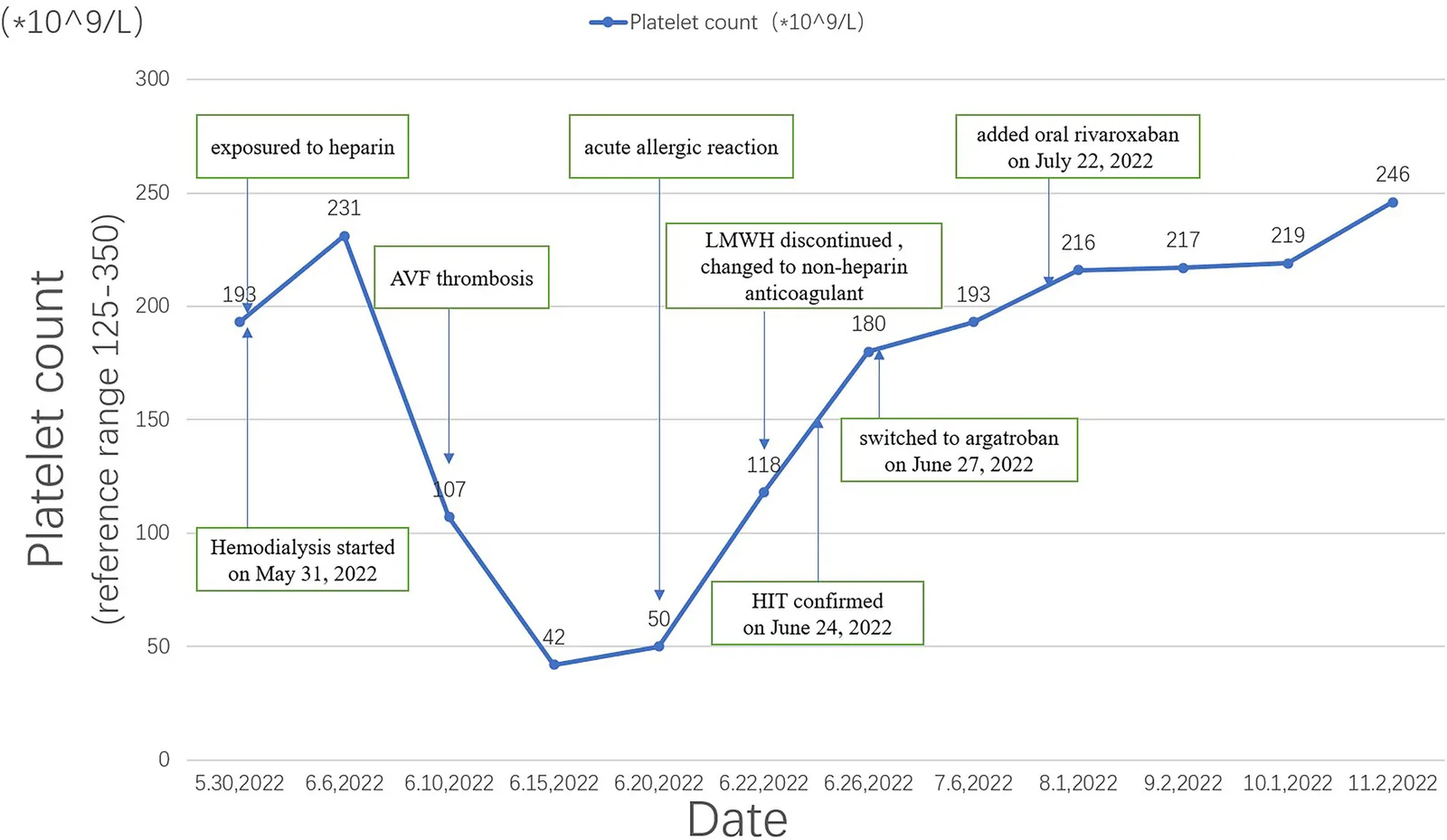
Time course of platelet count changes. AVF = arteriovenous fistula, HIT = heparin-induced thrombocytopenia, LMWH = low molecular weight heparin.

We describe the case of a 69-year-old female patient with a dry weight of 60 kg, who has a history of type 2 diabetes mellitus and hypertension. On May 30, 2022, she presented with worsening edema in both lower limbs, along with elevated serum creatinine levels. The renal function analysis showed a urea level of 33.18 mmol/L and a serum creatinine level of 920 µmol/L. The peripheral blood tests revealed white blood cell levels of 5.43 × 10^9^/L, platelet levels of 193 × 10^9^/L, and hemoglobin of 70 g/L. A renal ultrasound showed that both kidneys are atrophied, indicating chronic kidney disease. The patient was diagnosed with “chronic renal failure” and treated with hemodialysis, using low molecular weight heparin for anticoagulation, on May 31, 2022. Seven days after admission, an AVF shunt was created between the left radial artery and cephalic vein. Repeat blood tests showed hemoglobin of 82 g/L and platelet levels of 231 × 10^9^/L. On June 10, 2022 (the 10th day after exposure to heparin), blood tests revealed hemoglobin of 93 g/L and platelet levels of 107 × 10^9^/L. Surgical treatment was performed due to thrombosis and occlusion of the autogenous AVF in the left forearm. We successfully removed the thrombus from the fistula vein using a Fogarty catheter, and then we proceeded with the reconstruction of the autogenous AVF (Fig. 1). Due to clotting of the dialyzer and dialysis circuit during dialysis sessions, the dose of low molecular weight heparin was increased to 4100IU starting from June 13, 2022. The patient experienced tolerable and self-resolving chest discomfort on June 15, which was 15 days after exposure to heparin and 2 days after the increase in anticoagulant dosage, as well as on June 17. During hemodialysis on June 20, 2022, approximately 10 minutes after the treatment started, the patient experienced profuse sweating, chest tightness, shortness of breath, and hoarseness. Vital signs were recorded as follows: temperature 36.7°C, heart rate 90 bpm, respiratory rate 30 bpm, blood pressure 157/78 mmHg, and peripheral capillary oxygen saturation 86%. An acute allergic reaction was suspected. The patient received a 5 mg intravenous dose of dexamethasone, oxygen therapy via a nasal cannula, and hemodialysis was temporarily halted. Additionally, the dialyzer and blood circuit were replaced, and the extracorporeal circuit received a 1500 mL saline flush. Two minutes later, the patient’s symptoms improved, and vital signs were measured as follows: heart rate 82 bpm, respiratory rate 18 bpm, blood pressure 145/77 mmHg, and peripheral capillary oxygen saturation 97%. A complete blood count showed a decrease in platelet levels (50 × 10^9^/L) (Fig. 2). Approximately 1 hour later, the patient underwent re-dialysis without any anticoagulants.

The patient’s 4T predictive scoring system for thrombocytopenia (4T) score is 8 points, indicating a high risk for HIT. This score is calculated as follows: platelet count decrease >50% and platelet nadir ≥20 × 10^9^/L (2 points); platelet decrease on the 10th day after the start of heparin (2 points); new venous thrombosis (AVF thrombosis occlusion) and post-heparin bolus acute systemic reaction (2 points); and no alternative explanation for a decrease in platelet count (2 points). Based on the patient’s medical history and clinical presentation, there is a suspicion of HIT. Low molecular weight heparin was discontinued, and on June 22, 2022, citrate anticoagulation was initiated for hemodialysis, along with catheter locking. HIT-related IgG antibody was sent for testing. Recheck blood tests showed hemoglobin of 97 g/L and platelet levels of 118 × 10^9^/L. On June 24, 2022, the test result was positive for HIT-related IgG antibody with an optical density of 1.64 (enzyme-linked immunosorbent assay method, reference range <0.40). On June 26, 2022, follow-up blood tests indicated hemoglobin of 98 g/L and platelet levels of 180 × 10^9^/L (above 150 × 10^9^/L). Liver function tests indicated a total bilirubin level of 6.8 µmol/L, a direct bilirubin level of 1.2 µmol/L, an indirect bilirubin level of 5.6 µmol/L, an alanine aminotransferase level of 11 U/L, and an aspartate aminotransferase level of 16 U/L. On June 27, 2022, during hemodialysis, the anticoagulant was switched to argatroban injection, and 10% sodium chloride injection was used for catheter locking. On July 22, 2022, rivaroxaban tablets were added for oral anticoagulation (15 mg twice daily), and urokinase was utilized for catheter locking. Hemodialysis without argatroban was initiated on September 12, 2022. The dialysis process proceeded smoothly, and the patient experienced no significant discomfort. On January 18, 2023, vascular access for hemodialysis was converted to an autogenous AVF in the left forearm (Table 1).

**Table 1:** Timeline of case events.

Date	Event description
May 30, 2022	The patient presented with worsening lower limb edema and elevated serum creatinine and was diagnosed with chronic kidney failure.
May 31, 2022	Initiation of hemodialysis with LMWH for anticoagulation.
June 6, 2022	An arteriovenous fistula was created between the left radial artery and the cephalic vein.
June 10, 2022	Thrombosis and occlusion of the AVF in the left forearm. Platelet count was 231 × 10^9^/L.
June 13, 2022	Dose of LMWH increased to 4100 IU to address clotting in the dialysis circuit.
June 15, 2022	The patient experienced tolerable and self-resolving chest discomfort. Platelet count was 42 × 10^9^/L.
June 20, 2022	Acute allergic reaction during hemodialysis: profuse sweating, chest tightness, shortness of breath, and hoarseness. LMWH discontinued, treated with 5 mg intravenous dexamethasone and oxygen therapy. Platelet count was 50 × 10^9^/L.
June 22, 2022	LMWH discontinued. Citrate anticoagulation was initiated for hemodialysis and catheter locking. HIT antibody was sent for testing.
June 24, 2022	Positive result for HIT-related IgG antibody (optical density of 1.64).
June 26, 2022	Blood tests showed hemoglobin 98 g/L, platelet count 180 × 10^9^/L, liver function is normal.
June 27, 2022	Anticoagulation switched to argatroban. 10% sodium chloride injection was used for catheter locking.
July 22, 2022	Oral rivaroxaban (15 mg twice daily) was added for anticoagulation. Urokinase heparin was used for catheter locking.
September 12, 2022	Hemodialysis without argatroban was initiated.
January 18, 2023	Vascular access was converted to an AVF in the left forearm.

AVF = arteriovenous fistula, HIT = heparin-induced thrombocytopenia, LMWH = low molecular weight heparin.

## 3. Discussion and conclusion

HIT is a predominantly antibody-mediated adverse reaction that occurs during the use of heparin drugs. It is characterized by a decrease in platelet count and can also lead to venous and arterial thrombosis formation, such as deep vein thrombosis, pulmonary embolism, myocardial infarction, ischemic stroke, peripheral arterial thrombosis, and tissue necrosis of the limbs. In severe cases, it can even result in patient death. It is reported that approximately 50% of HIT patients will develop thrombosis, with a ratio of new venous thrombotic events to arterial thrombotic events being approximately 4:1.^[7,8]^ HIT can be categorized into 2 types: HIT-Ⅰ and HIT-Ⅱ.^[2]^ HIT-Ⅰ usually occurs within 2 days after exposure to heparin. It results in a mild decrease in platelet count, typically not falling below 100 × 10^9^/L and does not lead to thrombosis or bleeding. Platelet counts can recover spontaneously without stopping heparin or special treatment. This type of HIT may result from non-immune platelet aggregation caused directly by heparin and is not clinically significant. HIT-Ⅱ usually occurs 5 to 10 days after exposure to heparin. In most cases, platelet counts drop by more than 50% from the baseline, typically resulting in an absolute count lower than 150 × 10^9^/L, and generally do not fall below 20 × 10^9^/L. It can be accompanied by complications such as the formation of thrombosis and embolism. Some patients may experience acute systemic reactions, such as profuse sweating, chills, fever, chest tightness, dyspnea, hoarseness, tachycardia, hypertension or hypotension, and decreased blood oxygen saturation. This type of HIT is immune-mediated, and possible mechanisms are as follows^[1,9]^: Conformational changes occur when platelet factor 4 (PF4), released from activated platelet α-granules, binds to heparin, forming PF4-heparin complexes (PF4-H). This binding exposes new antigenic epitopes on PF4, stimulating B lymphocytes to produce anti-PF4-H antibodies (i.e., HIT antibodies). These IgG-type antibodies bind to PF4-H, forming IgG-PF4-H complexes, which then bind to Fcγ receptors on platelets (FcγRIIa). This aggregation triggers platelet activation and release of procoagulant microparticles, enhancing thrombin generation. Additionally, IgG-PF4-H complexes bind to Fc receptors on neutrophils (FcγRIIa), causing the release of extracellular traps that interact with platelets, red blood cells, PF4, and von Willebrand factor. They also bind to FcγRI on monocytes, triggering the release of tissue factor, which in turn activates the coagulation cascade and enhances thrombin production. Damage to vascular endothelial cells also releases tissue factor, significantly amplifying thrombin generation. The interaction of the above factors enhances platelet activation and aggregation and also increases thrombin generation, which contributes to the formation of thrombosis. The reticuloendothelial system, including the spleen, liver, and bone marrow, identifies and removes platelets bound by HIT IgG.^[10]^ Additionally, platelet consumption during thrombosis is another cause of platelet decrease. It is worth noting that infections, diabetes, kidney disease, trauma, and extracorporeal circulation in cardiopulmonary bypass can also lead to elevated PF4 levels.^[11]^

**Figure 3.**
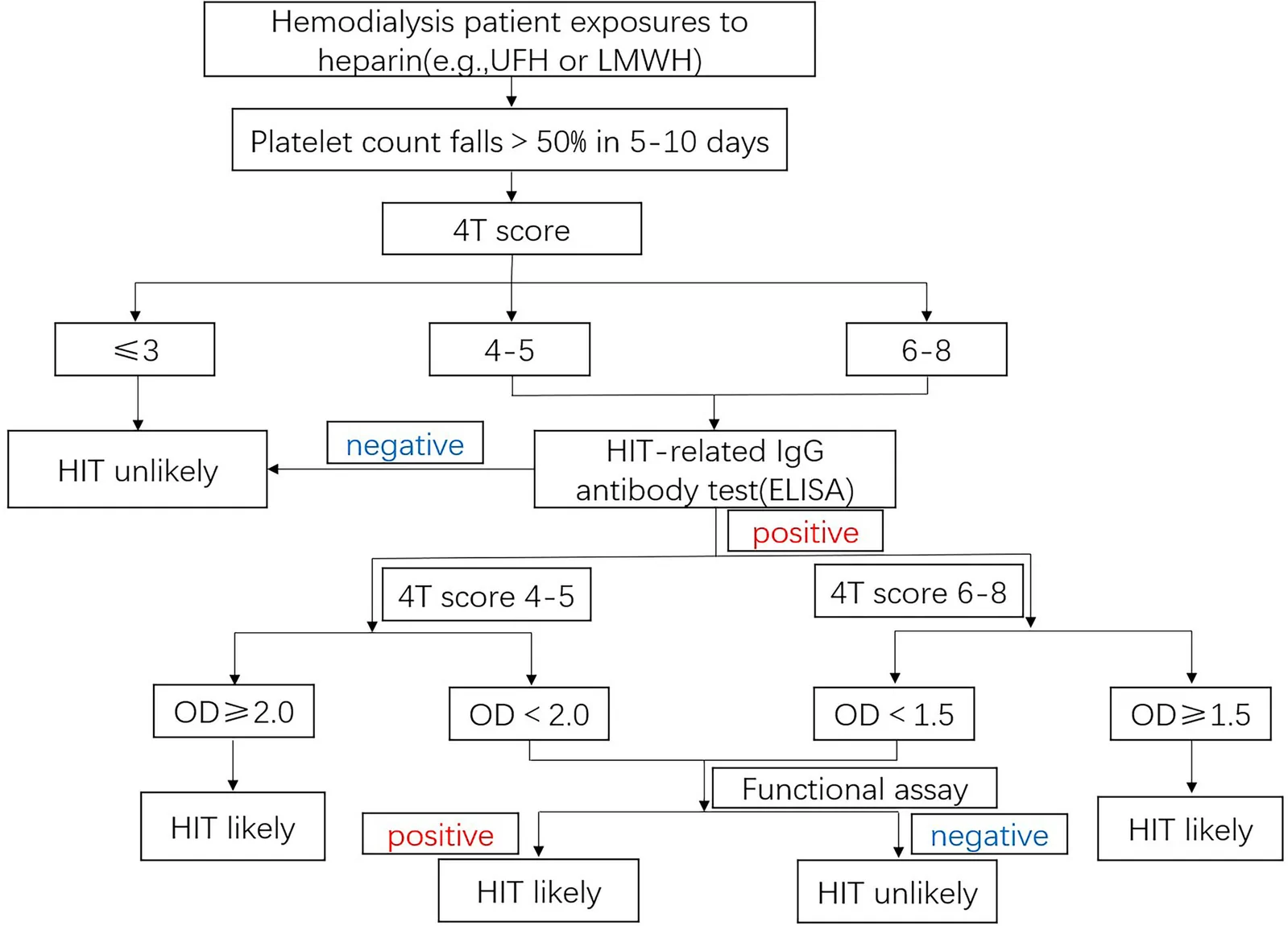
The flowchart of recommended approach to suspected HIT in hemodialysis patients. ELISA = enzyme-linked immunosorbent assay, HIT = heparin-induced thrombocytopenia, LMWH = low molecular weight heparin, OD = optical density, UFH = unfractionated heparin.

The diagnosis of HIT depends on a combination of a history of heparin exposure, clinical manifestations, and a significant decrease in platelet count, with or without thrombosis. It is also essential to differentiate HIT from conditions, such as thrombotic thrombocytopenic purpura, immune thrombocytopenic purpura, drug- or infection-induced thrombocytopenia, EDTA-induced platelet aggregation, etc. Currently, the standard diagnostic method for HIT involves the 4T’s along with dynamic monitoring of platelet count, in conjunction with HIT antibody testing and/or platelet function tests for exclusion and confirmation.^[12]^ The 4T’s system comprises 4 criteria: the degree of thrombocytopenia, the timing of platelet decline after heparin exposure, the presence of thrombosis or other sequelae from HIT, and the likelihood of alternative causes for thrombocytopenia.^[12]^ The 4T’s scores of ≤3, 4 to 5, and 6 to 8 signify a low (<1%), intermediate (approximately 10%), and high (approximately 50%) likelihood of HIT, respectively.^[13]^ If HIT is clinically suspected and the 4T score is 4 or higher, HIT IgG-specific antibody testing can be performed.^[13,14]^ A diagnosis can be confirmed under the following conditions: if the 4T score is between 4 and 5 and the HIT IgG-specific antibody is positive with an optical density of 2.0 or higher, or if the 4T score is between 6 and 8 and the HIT IgG-specific antibody is positive with an optical density of 1.5 or higher. If the optical density is below 2.0 (4T score 4–5) or 1.5 (4T score 6–8), further platelet function testing is needed for validation^[12]^ (Fig. 3). It is essential to recognize that a negative result for HIT IgG-specific antibody carries significant exclusionary value. When the HIT IgG-specific antibody test is negative, HIT can effectively be ruled out. Literature indicates that the prevalence of anti-HIT IgG-specific antibodies in patients undergoing maintenance hemodialysis ranges from 3.0% to 12.9%.^[15–19]^ This may be associated with their long-term exposure to heparin. However, only a small percentage of these patients develop HIT. Therefore, a positive result for anti-HIT IgG-specific antibody alone is not sufficient for diagnosing HIT.

Once HIT is confirmed, all medications containing heparin should be stopped immediately. In cases of thrombosis or embolism, thrombolytic agents such as urokinase or alteplase may be administered. Platelet counts typically start to rise 2 to 3 days after discontinuing heparin-containing medications and usually return to normal within 1 week.^[20]^ HIT IgG-specific antibodies remain in circulation for about 50 to 85 days, usually not exceeding 100 days.^[21,22]^ Meanwhile, reports indicate that HIT IgG antibodies can persist in patients for extended periods, with some remaining positive for 1.5 to 4 years.^[23,24]^ Readministering unfractionated heparin or low molecular weight heparin within 100 days after the onset of HIT may trigger acute systemic allergic reactions. Therefore, if there is a moderate to high suspicion of HIT, unfractionated heparin or low molecular weight heparin should be immediately discontinued. Additionally, the use of heparin for pre-dialysis circuits and dialyzer priming should be avoided. There is ongoing controversy regarding readministration of heparin after the HIT antibodies test negative. The ACCP guidelines recommend short-term use of heparin for patients undergoing cardiac surgery. However, for patients undergoing percutaneous coronary intervention treatment, non-heparin anticoagulants are preferred.^[22]^ For patients undergoing hemodialysis, we do not recommend readministering heparin after HIT antibodies have tested negative. This is due to the high risk of acute systemic allergic reactions that may occur upon readministration. Fortunately, several non-heparin anticoagulants are clinically available and can serve as effective alternatives. For patients with refractory HIT, particularly those who experience slow platelet recovery after switching to non-heparin anticoagulant therapy, or who are in a severe hypercoagulable state or have recurrent ischemic strokes, intravenous immunoglobulin or plasma exchange may be a treatment option.^[25]^ A common dosage is 0.4 g/kg per day for 5 days or 1.0 g/kg per day for 2 days.^[26,27]^ This approach has the potential to achieve favorable clinical outcomes, and initiating treatment early, ideally within 7 to 14 days after switching to non-heparin anticoagulants, may help reduce the sequelae.^[26]^

Unfractionated heparin and low molecular weight heparin are the most commonly used anticoagulants for maintaining the patency of filters and extracorporeal circuits during hemodialysis therapy. The incidence of HIT in hemodialysis patients receiving heparin for anticoagulation is highest during the initial phase and significantly decreases during the maintenance phase.^[15]^ The exact incidence of HIT in patients with end-stage renal disease is not well-defined. Kaur et al reported an incidence rate of 4.6% among end-stage renal disease patients receiving heparin during hospitalization.^[28]^ Similarly, Yamamoto et al found that 3.9% of hemodialysis patients were diagnosed with HIT within 59 days of starting regular heparin anticoagulation for their treatment.^[17]^ Mya et al suggested a lower incidence rate of 1.0% among hemodialysis patients using unfractionated heparin for anticoagulation.^[29]^ Additionally, research conducted by Doi et al indicated an incidence rate of 0.6% among hemodialysis patients treated with low molecular weight heparin for anticoagulation.^[30]^ For patients undergoing hemodialysis who have HIT, sodium citrate or argatroban is recommended as an alternative anticoagulant. Argatroban is primarily metabolized by the liver, so no dose adjustment is necessary for patients with renal impairment. However, patients with hepatic impairment will require a dose reduction. Intermittent renal replacement therapy can be performed without a loading dose.^[31]^ In patients with normal liver function, maintenance infusion rates should not exceed 10 µg/kg/min, starting at 2 µg/kg/min.^[3,14]^ For patients with hepatic dysfunction (e.g., serum total bilirubin ≥25.6 µmol/L), heart failure, severe anasarca, or those who have undergone post-cardiac surgery, the initial infusion rate should be between 0.5 and 1.2 µg/kg/min.^[14]^ Coagulation function should be assessed at the end of each 4-hour dialysis session. The dosage for the next treatment should be adjusted based on the activated partial thromboplastin time (APTT) level. The target APTT levels should be 1.5 to 3.0 times the baseline value, without exceeding 100 seconds.^[14]^ For critically ill patients with multi-organ dysfunction requiring continuous renal replacement therapy, a loading dose of 100 μg/kg should be administered. Following this, a maintenance infusion of argatroban can be started at a rate of 0.5 μg/kg/min. The dosage should be adjusted every 2 to 4 hours based on APTT levels, aiming to maintain APTT levels at 1.5 to 3.0 times the baseline value.^[32,33]^

When hemodialysis patients with HIT are stabilized and their platelet counts have recovered to baseline levels or ≥150 × 10^9^/L, transitioning to warfarin or other novel oral anticoagulants (such as dabigatran, rivaroxaban, apixaban, and edoxaban) may be considered.^[20,22]^ Warfarin has a slow onset of action, typically taking 3 to 5 days to exert its anticoagulant effect. Therefore, it is recommended to overlap its use with non-heparin anticoagulants for at least 5 days when starting warfarin therapy.^[34]^ The initial dose should be no more than 3 mg per day. Regular monitoring of the international normalized ratio is necessary, with a target range of 2.0 to 3.0. Novel oral anticoagulants offer the advantage of convenient administration without the need for regular international normalized ratio monitoring. Among these agents, rivaroxaban has the most extensive application experience in patients with HIT,^[35,36]^ with many studies reporting its safety and effectiveness.^[37–40]^ Studies have found that rivaroxaban can further alleviate decreases in platelet counts and reduce the incidence of thrombotic events in HIT patients.^[36]^ The recommended initial dose of rivaroxaban is 15 mg, taken twice daily. Once normalization of platelet counts or 21 days after the occurrence of acute thrombotic events, the dose should be reduced to 20 mg once daily.^[37]^ Aly et al^[41]^ reported a case involving a patient on maintenance hemodialysis with a history of HIT who experienced a pulmonary embolism after contracting COVID-19. The patient was treated with apixaban at a dosage of 2.5 mg twice daily, leading to satisfactory outcomes over 3 months. Additionally, Trujillo Agudelo et al^[42]^ documented a case of a patient with chronic kidney disease who was diagnosed with acute limb ischemia of the right leg secondary to an occlusive thrombus of the femoral artery and underwent a surgical embolectomy. This patient developed HIT following treatment with heparin after surgery, treated with apixaban, also at 2.5 mg twice daily, resulting in gradual recovery of platelet counts without any further thrombotic events. We systematically searched the relevant literature in the databases and reviewed a total of 6 papers,^[7,23,31,43–45]^ including 10 patients; the basic information, thrombotic events, and treatment are shown in Table 2.

**Table 2:** Literature review of type Ⅱ heparin-induced thrombocytopenia in hemodialysis patients.

References	Age/sex	Kidney disease	Type of heparin	OD value (U/mL)	4T score	Onset of HIT	Nadir platelet (×10^9^/L)	Thrombotic events	Therapy
Lim et al^[43]^	80/F	CKD	UF	7.63	8	7 days after the start of hemodialysis	15	Acute thrombotic stroke	Argatroban and warfarin
Gameiro et al^[31]^	82/F	CKD	UF	+	5	15 days after the start of hemodialysis	33	None	Warfarin, citrate lock
	83/F	CKD	UF	+	7	7 days after the start of hemodialysis	12	Deep venous thrombosis, pulmonary embolism, circuit coagulation	Warfarin, citrate lock
	73/F	AKI-on-CKD	UF	+	7	5 days after the start of hemodialysis	100	Circuit coagulation	Warfarin, citrate lock, transition to peritoneal dialysis
	85/M	AKI	UF	+	4	7 days after the start of hemodialysis	34	None	Warfarin, citrate lock
	77/M	AKI	UF	+	4	4 days after the start of hemodialysis	74	None	Bivalirudin, citrate lock
Wang et al^[44]^	73/M	CKD	UF and LMWH	7.1	7	7 days after exposure to heparin	45	Acute cerebral infarction, circuit coagulation	Bivalirudin
Furuto et al^[45]^	76/M	AKI	UF	5.6	6	14 days after the start of hemodialysis	47	Cerebral infarction, clotting in the hemodialysis catheter	Argatroban
Murakami et al^[23]^	64/M	AKI	UF	2.648	7	1 day after exposure to heparin	78	Circuit coagulation	Nafamostat mesylate and argatroban
Nishiyama and Higashi^[7]^	84/F	CKD	LMWH	5.1	5	4 years after the start of hemodialysis	27	None	Nafamostat mesylate and argatroban

4T = 4T predictive scoring system for thrombocytopenia, AKI = acute kidney injury, CKD = chornic kidney disease, F = female, LMWH = low molecular weight heparin, M = male, OD = optical density, UF = unfractionated heparin, + = positive.

In summary, we reported a case involving a hemodialysis patient who developed thrombosis in the left forearm AVF on the 10th day following initial exposure to low molecular weight heparin, alongside concomitant thrombocytopenia. On the 20th day of hemodialysis, the patient experienced an acute systemic allergic reaction, which resulted in a decrease in platelet count of over 50% compared to baseline. Her 4T score was 8, and the HIT IgG-specific antibody was positive, with an optical density value of 1.64, confirming the diagnosis of HIT. After adjusting the anticoagulant therapy to argatroban injection, the patient’s platelet count recovered to near baseline levels within approximately 1 week, and no further symptoms related to acute systemic reactions occurred. The patient initially presented with thrombosis in the vascular access, and HIT was not suspected until she experienced an acute systemic allergic reaction and a significant drop in platelet count during hemodialysis. This case highlights the importance of considering the possibility of HIT in hemodialysis patients, particularly during the induction phase, when new thrombosis occurs in the vascular access and is accompanied by a decrease in platelet count. Early diagnosis, followed by a transition to non-heparin anticoagulants, can help reduce the risk of more severe symptoms in patients. In addition, hemodialysis can be safely performed using only oral rivaroxaban.

## 4. Limitations

The limitation of this case report is that we did not continuously monitor changes in HIT antibody titers after stopping low molecular weight heparin. Continuous monitoring could have helped us better understand the patient’s clinical progression. In the future, we will improve our tracking of HIT antibody titers to better understand this condition in such patients.

## Author contributions

**Conceptualization:** Xiaming Zhang.

**Data curation:** Xiaming Zhang, Mi Zhou, Ping Wu, Yingji Chen.

**Formal analysis:** Xiaming Zhang.

**Supervision:** Juzhen Yan.

**Writing – original draft:** Xiaming Zhang.

**Writing – review & editing:** Xiaming Zhang, Juzhen Yan.
